# Non-Invasive Molecular Imaging of Fibrosis Using a Collagen-Targeted Peptidomimetic of the Platelet Collagen Receptor Glycoprotein VI

**DOI:** 10.1371/journal.pone.0005585

**Published:** 2009-05-18

**Authors:** Julien Muzard, Laure Sarda-Mantel, Stéphane Loyau, Alain Meulemans, Liliane Louedec, Claudie Bantsimba-Malanda, Florence Hervatin, Joëlle Marchal-Somme, Jean Baptiste Michel, Dominique Le Guludec, Philippe Billiald, Martine Jandrot-Perrus

**Affiliations:** 1 INSERM, U698, Hôpital Bichat, Paris, France; 2 INSERM, U773, CRB3, Faculté Xavier Bichat, Paris, France; 3 Museum National d'Histoire Naturelle, CNRS-FRE 3206, Paris, France; 4 INSERM, U700, Faculté Xavier Bichat, Paris, France; 5 Université Paris7, Paris, France; 6 AP-HP, Hôpital Bichat, Paris, France; Harvard Medical School, United States of America

## Abstract

**Background:**

Fibrosis, which is characterized by the pathological accumulation of collagen, is recognized as an important feature of many chronic diseases, and as such, constitutes an enormous health burden. We need non-invasive specific methods for the early diagnosis and follow-up of fibrosis in various disorders. Collagen targeting molecules are therefore of interest for potential *in vivo* imaging of fibrosis. In this study, we developed a collagen-specific probe using a new approach that takes advantage of the inherent specificity of Glycoprotein VI (GPVI), the main platelet receptor for collagens I and III.

**Methodology/Principal Findings:**

An anti-GPVI antibody that neutralizes collagen-binding was used to screen a bacterial random peptide library. A cyclic motif was identified, and the corresponding peptide (designated collagelin) was synthesized. Solid-phase binding assays and histochemical analysis showed that collagelin specifically bound to collagen (Kd 10^−7^ M) *in vitro*, and labelled collagen fibers *ex vivo* on sections of rat aorta and rat tail. Collagelin is therefore a new specific probe for collagen. The suitability of collagelin as an *in vivo* probe was tested in a rat model of healed myocardial infarctions (MI). Injecting Tc-99m-labelled collagelin and scintigraphic imaging showed that uptake of the probe occurred in the cardiac area of rats with MI, but not in controls. *Post mortem* autoradiography and histological analysis of heart sections showed that the labeled areas coincided with fibrosis. Scintigraphic molecular imaging with collagelin provides high resolution, and good contrast between the fibrotic scars and healthy tissues. The capacity of collagelin to image fibrosis *in vivo* was confirmed in a mouse model of lung fibrosis.

**Conclusion/Significance:**

Collagelin is a new collagen-targeting agent which may be useful for non-invasive detection of fibrosis in a broad spectrum of diseases.

## Introduction

Collagen, a major component of the extracellular matrix (ECM), is one of the determinants of tissue structure. Fibrosis is characterized by the pathological accumulation of collagen, and is increasingly recognized as an important feature of many chronic diseases, and as such, represents an enormous health burden [1]. It is estimated that 45% of deaths in the United States can be attributed to conditions associated with fibrosis. In the absence of a non-invasive specific marker, the only method available for quantifying fibrosis is tissue biopsy, which is invasive and carries a risk of complications in a variety of organs and cannot be easily repeated. Functional tests are currently used to assess the degree to which organs are affected, but functional impairment only occurs in the presence of a relatively high degree of fibrosis. This means that we still need non-invasive specific methods for the early diagnosis and follow-up of fibrosis in many disorders in which fibrosis is of major prognostic interest. For this purpose, quantitative imaging methods have the advantage over blood biomarkers of being able both to quantify and localize the fibrotic process. Recent studies have shown that transient echography or MRI elastography provide ways to assess liver fibrosis by non-invasively measuring liver stiffness in adult patients [2], [3]. Preliminary experiments have also been performed using diffusion-weighted MRI to quantify liver fibrosis [4]. However, these techniques are not specific for fibrosis and may suffer from a lack of sensitivity, high levels of fibrosis being necessary before tissue elasticity and diffusion properties are impaired. Recently, molecular imaging of cardiac fibrosis was reported using radiotracers specific for targets co-expressed or co-located with fibrosis in patients and mice with post-infarction cardiomyopathy: 18F-fluorobenzoyl-lisinopril specific for angiotensin-converting enzyme [5], Tc-99m losartan specific for angiotensinII receptors [6], 99mTc-Cy5.5 RGD imaging peptide targeting proliferating myofibroblats [7], [8]. However such indirect tracers are not adapted to all clinical situations involving fibrosis, because of different physiopathology and the need to detect fibrosis as well as fibrogenesis. Specific and direct tracers for the molecular imaging of fibrosis, especially collagen-targeting molecules, constitute a challenge and a potentially wide field of interest for imaging methods, including radionucleide imaging and MRI [9].

The inherent collagen binding properties of the collagen receptors should make them good models for developing collagen probes. Collagen receptors interact with the triple helical structures of collagen fibrils [10]. Several members of the integrin family, including the alpha1beta1, alpha2beta1 and alpha1beta1 integrins, are widely expressed collagen receptors, but since they also bind to other matrix proteins, they are not suitable for specifically targeting collagen. The immunoadhesin glycoprotein VI [11], [12] has good affinity and high specificity for type-I and type-III collagens and has been extensively characterized. GPVI seems to be an attractive target for the development of collagen probes. Soluble recombinant GPVI has even been proposed as a tool for *in vivo* imaging of collagen exposed by unstable atherosclerotic plaques. However, an efficient collagen probe must be small enough to gain access to the interstitial space. We therefore decided to focus on peptides that mimicGPVI, and have taken advantage of a monoclonal antibody, 9O12.2, which binds GPVI with a high affinity, and neutralizes the interaction between GPVI and collagen *in vitro* and *in vivo* [13], [14]. We hypothesized that the 9O12.2 epitope must, at least in part, overlap with the collagen binding-site on GPVI. Using a bacterial display approach, a peptidomimetic of GPVI has been identified. This peptide, designated collagelin, exhibits collagen-binding properties both *in vitro* and *ex vivo*. The suitability of collagelin as a probe for the molecular imaging of fibrosis is assessed *in vivo* by isotopic imaging of scars in a rat model of healed myocardial infarction and a mouse model of lung fibrosis.

## Results

### Identification of 9O12.2-binding peptides

**Figure 1:**
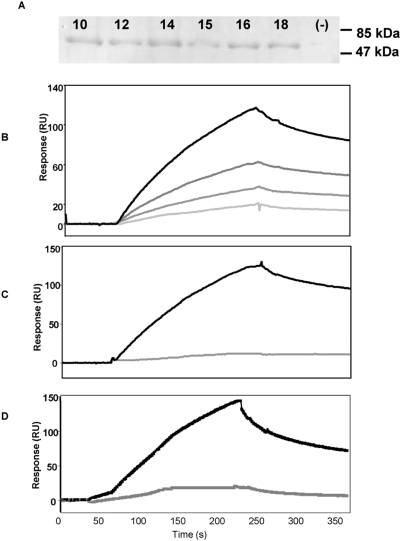
Identification of collagelin. A: *Identification of 9O12.2 binding bacterial clones*. Proteins from bacterial clones were separated by electrophoresis under non-reducing conditions, and analysed by immunoblot using the 9O12.2 IgG. The band at ∼63 kDa corresponds to the FliTrx fusion protein containing a peptide recognized by 9O12.2. Results are from six selected clones (10, 12, 14, 15, 16, 18), and from one clone selected from the same library but using an irrelevant antibody (−). The sequence of clone 14 was retained for peptide synthesis. B–D *Surface plasmon resonance (SPR) analysis of collagelin binding to 9O12.2*. In B, increasing concentrations of the 9O12.2 IgGs were passed over the sensorchip (4, 6, 8, 10 µg/ml from bottom to top). In C: 9O12.2 IgG (8 µg/ml) was injected over immobilized B-collagelin that was either non-reduced (black) or reduced by DTT (gray) on the sensorchip. In D, 9O12.2 IgG (5 µg/ml) was injected over immobilized B-collagelin in the absence (black) or presence of recombinant soluble GPVI (25 µg/ml) (gray). Representative sensorgrams are shown after subtracting the non-specific response from a control flow cell coated with an irrelevant peptide.

After five rounds of panning the combinatorial bacterial peptide library using 9O12.2 IgG, 20 clones were selected that produced a flagellar fusion protein recognized by 9O12.2 on immunoblots (Figure 1A). DNA sequencing of all the inserts showed some redundancy and identified 9 peptide sequences (Table 1) with 7 common residues and differing from each other by one to 5 residues. None of these sequences was registered in any database.

**Table 1:** (A): Amino-acid alignment (Fasta format) of the 20 clones sequenced after screening of the FliTrx random peptide display library against immobilized 9O12 IgG.

clones 1–10	RFMHGLQLWADE
clone11	RVMHGLQLWADE
clones12–13	RVMHGLQLWADE
clone14	RVMHGLHLGDDE
clone15	RVMHGLHLWDDE
clone16	RVMHGLQLWDDE
clone17	RVMHGLHLWADE
clone18	FVMHGLHLGDDE
clone19	PVMHGLHLWDDE
clone20	RVMHGLLLGADE

The sequence of the peptide that has been selected for synthesis is underlined.

Sequence 14, RVMHGLHLGDDE (single letter amino acid code), was selected for synthesis. A constrained peptide, designated collagelin, was synthesized and conjugated with biotin (B–collagelin). The molecular masses of the peptide and the conjugate (2155 and 2404 Da respectively) and their purity (>95%) were determined by mass spectrometry.

Surface plasmon resonance experiments showed that 9O12.2 IgG bound to immobilized B–collagelin in a dose-dependent manner (Figure 1B) with a K_D_ of 10^−6^ M. The 9O12.2 IgG did not bind to reduced collagelin (Figure 1C), confirming that the 9O12.2 epitope is conformational [13]. Binding of 9O12.2 IgG to collagelin was inhibited in the presence of soluble GPVI (Figure 1D), indicating that GPVI and collagelin competed for binding to 9O12.2.

### Binding to collagen

**Figure 2:**
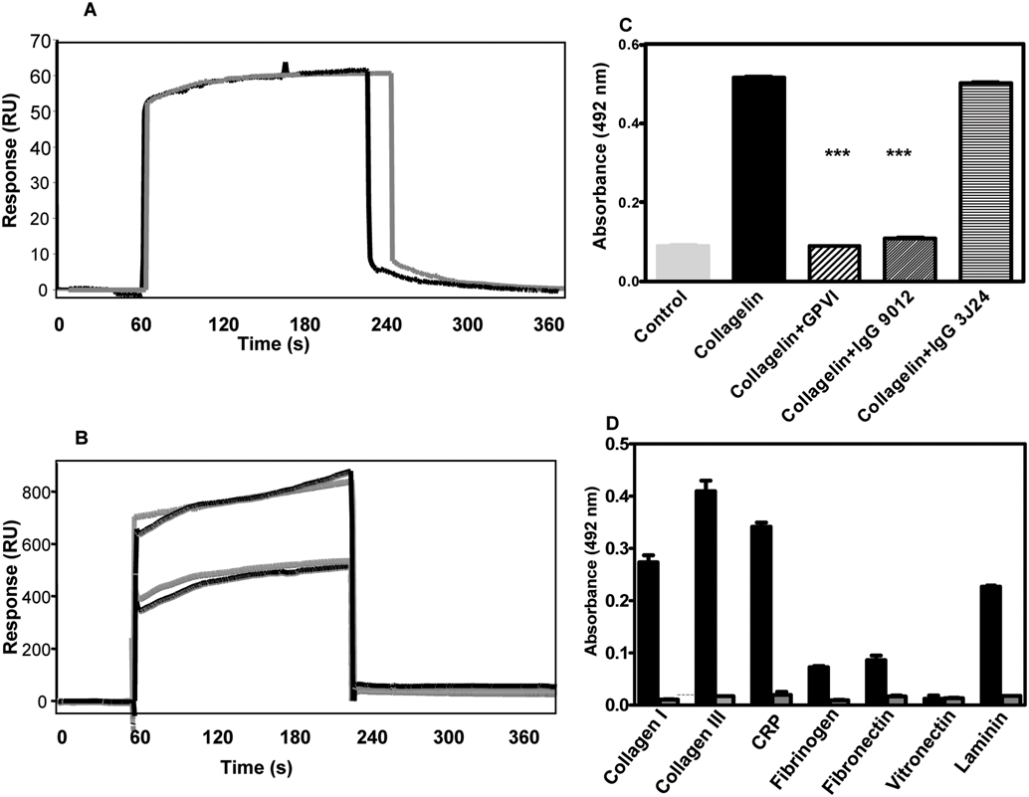
Interaction of collagelin with collagen. A: B-collagelin was immobilized on a streptavidin-coated sensorchip (∼20 RU). Collagen (10 µg/ml) was injected over the sensorchip. A representative sensorgram (dark line) and interaction fit (gray line) are shown after subtracting the non-specific background signal from a control flow cell coated with an irrelevant peptide. B: B-collagelin (250, 500 µg.mL^−1^) was injected over a collagen-coated sensorchip. Sensorgrams (black) and interaction fits (gray) are shown. Representative sensorgrams are shown after subtracting the non-specific response from the irrelevant peptide. C: B-collagelin or control peptide (50 µg. mL^−1^) were incubated with immobilized, fibrillar, type-I collagen in microtitration plates, and detected using HRP-coupled extravidin. In competition experiments, collagelin was mixed with GPVI-Fc (50 µg.mL^−1^), 9012.2 IgGs (50 µg.mL^−1^) or 3J24.2 IgGs (50 µg.mL^−1^) before being added to collagen-coated wells. Means±SD (n = 3) are presented; *** p<0.01. D: B-collagelin (50 µg.mL^−1^, black) or B-Pc (gray) were incubated with immobilized collagen I or III, CRP, fibrinogen, fibronectin, vitronectin and laminin in microtitration plates, and detected as above. Means±SD (n = 3) are shown.

Since collagelin mimicked the 9O12.2epitope, at least in part, it was assumed that it also mimicked the collagen binding site of GPVI. Using SPR, type I collagen was found to bind to a B-collagelin-coated sensorchip (Figure 2A). Furthermore, B-collagelin bound to a sensorchip coated with fibrillar type I collagen in a dose-dependent manner with a K_D_ of 1.10^−7^ M. (Figure 2B). In contrast, a non-relevant biotinylated peptide (B-Pc), did not bind to collagen. B–collagelin also bound to collagen-coated plates, and this binding was completely inhibited in the presence of GPVI-Fc or of 9O12.2 IgGs (Figure 2C), whereas the anti-GPVI monoclonal antibody 3J24.2, which binds to an epitope distinct from 9O12.2 [15], did not inhibit B-collagelin interaction with collagen. These results demonstrate that collagelin and GPVI bind to sites on collagen that must overlap or be identical.

The binding specificity of collagelin was determined using the GPVI specific ligand, collagen related peptide (CRP), and various different macromolecules from the extracellular matrix. The non-relevant peptide (Pc) did not bind to any of these proteins. Collagelin bound to type III collagen and to CRP, confirming its GPVI-like specificity. The binding of collagelin to vitronectin fibrinogen or fibronectin was found either to be weak or not to occur at all (Figure 2D). In contrast, collagelin consistently bound to laminin, previously identified as a GPVI accessory ligand [16]; the sites of GPVI that interact with laminin and collagen thus share a common structure that is mimicked by collagelin. Nevertheless, SPR analysis indicated that the affinity of collagelin for laminin was two orders lower than for collagen (K_D_ of 1.83 10^−5^ M).

### Ex vivo labeling of collagen with collagelin

**Figure 3:**
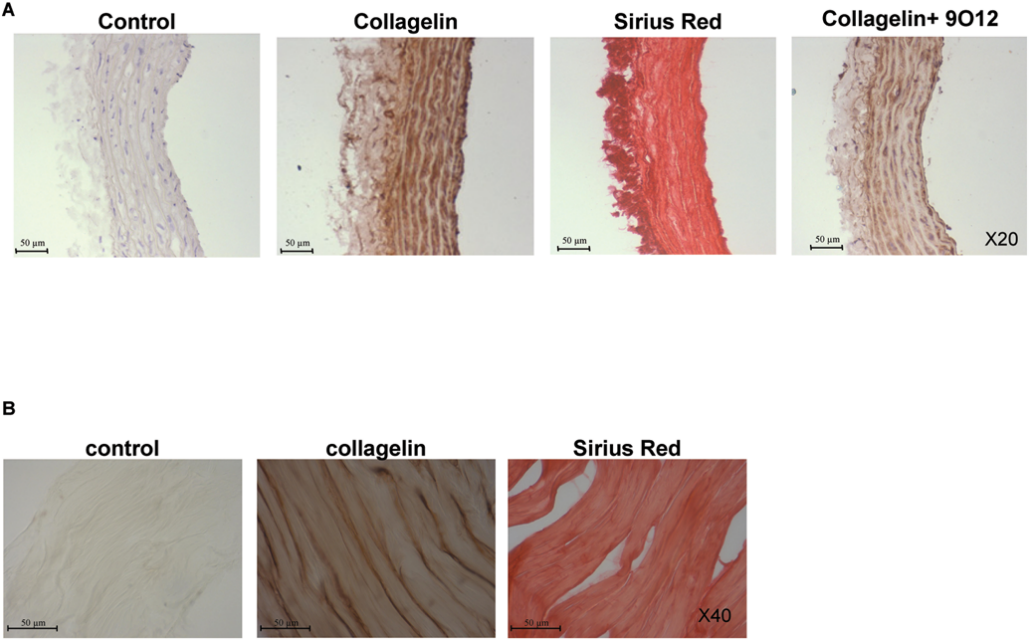
Histochemical analysis of peptide binding to tissue collagen. A: Frozen sections of rat aorta were incubated with B-collagelin or control peptide (200 µg/mL) and detected using HRP-coupled streptavidin. Sections were counter-stained with hematoxylin. Contiguous serial sections were stained with picrosirius red. In a competition experiment, the peptide was mixed with anti-GPVI IgG 9O121.2 (300 µg/mL). B: Paraffin embedded sections of rat tail tendon were treated as above.

The capacity of collagelin to interact with collagen was analyzed histochemically on frozen sections of rat aorta and tail tendon. Positive and specific brown staining developed when sections were treated with B-collagelin, but not with B-Pc. The staining produced by collagelin coincided with that of collagen stained with picrosirius red in serial sections (Figure 3). The intensity of the staining by collagelin decreased in the presence of 9O12.2 IgG, confirming the specificity of the interaction.

### In vivo isotopic molecular imaging of fibrosis

Collagelin was evaluated as a potential probe for fibrosis *in vivo* in rats. A model of healed myocardial infarction was used so that the uptake of the probe in healthy tissues could be compared to that in the collagen-rich fibrotic scar formed after myocardial remodeling. Imaging was performed at least three weeks after MI after the acute inflammatory phase and when the scar is well formed.

**Figure 4:**
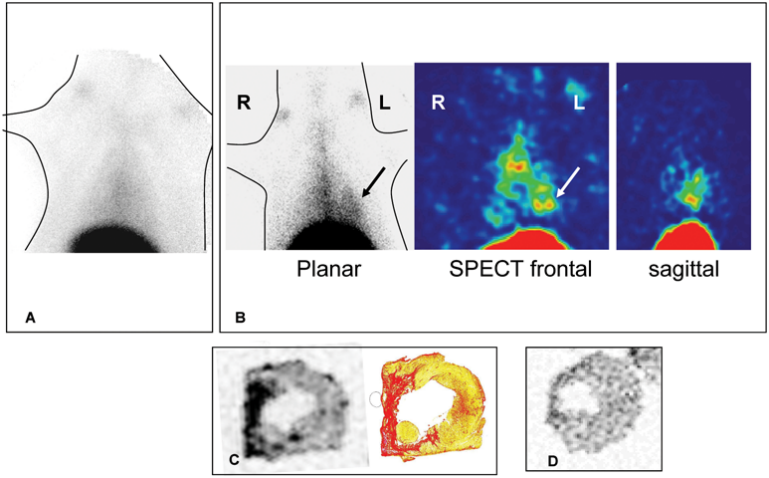
*In- vivo* scintigraphy, *ex vivo* myocardial autoradiography and histology using ^99m^Tc-streptavidin-B-collagelin. A: Planar thoracic scintigraphy of a control rat (sham). B: Planar and tomographic (frontal and sagittal views) thoracic images of a rat with a fibrotic myocardial infarction: a hot-spot (arrows) can be seen in the left ventricular myocardial area. C: Corresponding myocardial autoradiography and histology (collagen-specific picrosirius red staining,), confirming tracer uptake in the thin, fibrotic (red) myocardial scar (arrows). D: Control experiment: no activity can be seen in the myocardial scar of a rat injected with irrelevant ^99m^Tc-streptavidin-B-Pc.

First, B-collagelin and B-Pc were mixed with ^99m^Tc-streptavidin. *Ex vivo*, the uptake of ^99m^Tc-streptavidin-coupled-collagelin by rat aortic collagen was three times greater than the uptake of the control peptide Pc (p<0.001), and the uptake of labeled collagelin was inhibited by 9012.2 IgG. After intravenous injection of 1 nmol (70 MBq) to rats, SPECT imaging revealed high uptake by the liver, and slow blood clearance. Visual analysis of planar and SPECT images 4 h–6 h post injection revealed significant uptake of ^99m^Tc-streptavidin-collagelin in the cardiac area of all rats with an MI scar (n = 6), whereas no tracer uptake was observed in the cardiac area of sham-operated rats (n = 6) (Figures 4A–4B). Significant tracer uptake was also observed in the thoracotomy scar in 2 out of 6 rats with MI, and in 2 of 6 sham-operated rats. Mean heart-to-lung ratios which represent the contrast between the target and the surrounding tissues, were calculated on planar images as being of 2.76±0.36 and 1.95±0.28 for MI and sham-operated rats respectively (p<0.01). Histological analysis was performed on frozen sections of the myocardium obtained 6–8 hours after the injection. Autoradiography showed that the areas labeled by ^99m^Tc-streptavidin-collagelin superimposed on the collagen fibers of the infarct scars stained by picrosirius red (Figure 4C). In contrast, there was little or no uptake of the non-binding peptide Pc (Figure 4D). Mean infarct-to-remote myocardium radioactivity ratios were of 2.52±0.20 and 1.82±0.32 with collagelin and Pc respectively (p<0.006). Any fibrotic adhesions in contact with the myocardial scar (n = 1), concentrated high levels of the radiotracer.

**Figure 5:**
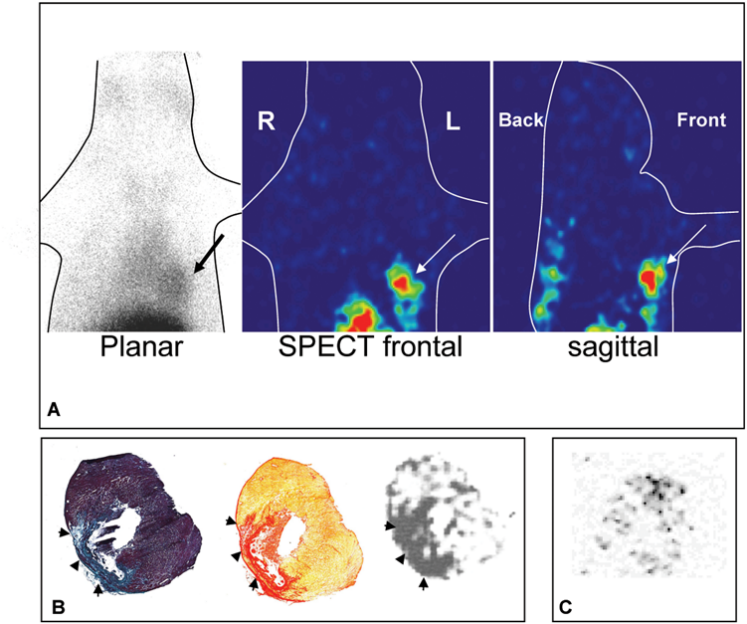
*In vivo* scintigraphy, *ex vivo* myocardial autoradiography and histology using 99mTc-collagelin. A: Planar thoracic scintigraphy of a rat with fibrotic myocardial infarction: a clear hot-spot (arrows) xcan be seen in the left ventricular myocardial area. B: From left to right, corresponding myocardial histology (Masson's trichrome, picrosirius red) and autoradiography, confirming tracer uptake in the thin, fibrotic (red) myocardial scar (arrow heads). C: Control experiment: very low activity is observed in the myocardial infarction in a rat injected with irrelevant 99mTc-Pc.

In a second set of experiments, collagelin and Pc were directly labeled with Tc-99m to avoid the drawbacks of high, non-specific liver uptake and slow blood clearance which could be attributed to streptavidin, and impaired the target-to-background ratio of the images. Radiolabeling efficiency exceeded 96% at 30 min, and exceeded 90% at 6 h, indicating high labeling stability. *Ex vivo* incubation of myocardial sections in the presence of radiotracers demonstrated that ^99m^Tc-collagelin accumulated in the scarred tissue, whereas the uptake of ^99m^Tc-Pc was significantly lower (p<0.05). After intravenous injection (1 nmol, 70 MBq) in rats, ^99m^Tc-collagelin demonstrated fast blood clearance (half life: 5±0.5 min), very early (<5 min) biliary excretion, low liver uptake (0.36% of injected dose per gram of tissue), fast digestive excretion occurring as soon as 10 min post-injection, and low renal uptake (n = 6). No thyroid or gastric uptake was observed until 6 h post-injection, suggesting that the radiolabeling was very stable *in vivo*. Figure 5A shows representative planar and SPECT images acquired two hours after injection, which reveal a significant uptake of the probe in the cardiac area of all but one of the rats with MI scars (n = 8). No tracer uptake was observed in the cardiac area of sham-operated rats (n = 6). Heart-to-lung ratios calculated on planar images were 2.08±0.17 and 1.61±0.23 in MI and sham-operated rats respectively (p<0.01). Comparison of autoradiography and histology findings for frozen myocardial sections showed that the ^99m^Tc-collagelin coincided with the collagen fibers of the scars (Figure 5B). There was little or no uptake of Pc by the scar (Figure 5C). Mean infarct-to-remote myocardium activity ratios were 2.92±0.53 and 1.83±0.3 for collagelin and Pc respectively (p<0.008). Any fibrotic adhesions in contact with the myocardial scar (n = 3) also concentrated high levels of ^99m^Tc-B-collagelin.

**Figure 6:**
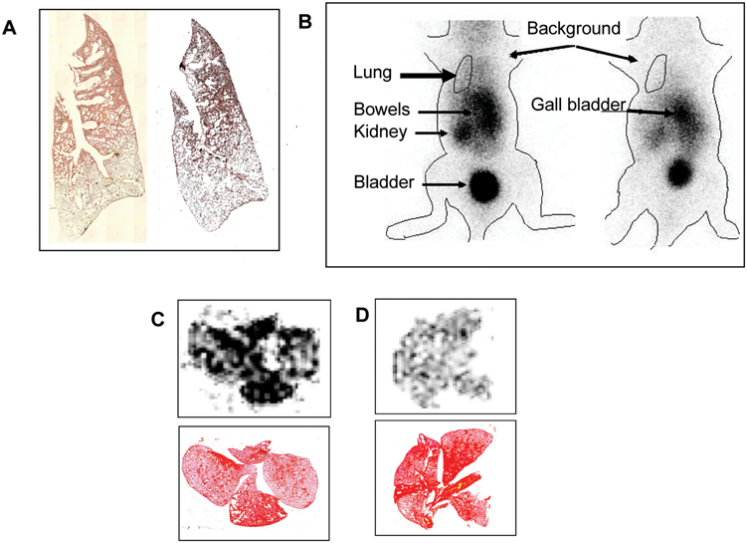
Imaging of lung fibrosis. A: Paraffin embedded sections of lungs from a bleomycin-treated mice were incubated with B-collagelin and detected using HRP-coupled streptavidin. Sections were counter-stained with hematoxylin. Contiguous serial sections were stained with picrosirius red. Representative images are shown. B: Whole body planar scintigraphy of a bleomycin-treated mouse (left) and a control mouse (right). Representative images acquired one hour after the injection of 99mTc-collagelin are shown. C: Corresponding lung autoradiography and histology (picrosirius staining) showing the colocalisation of 99mTc-collagelin with fibrosis. D: Lung autoradiogaphy and histology from a mice treated with the control 99mTc-Pc.

In order to confirm the specificity of imaging with collagelin, the tracer was tested in a mouse model of lung fibrosis. First, an histochemical analysis was performed on sections of healthy and fibrotic lungs (fig 6A). Staining by collagelin coincided with the collagen rich lesions. *In vivo* imaging was next realized 14 days after instillation of bleomycin. One hour after injection of ^99m^Tc-B-collagelin, scintigraphic imaging showed the uptake of the tracer in the pulmonary area of the mice that received bleomycin as compared to control mice (fig 6B) with lung to muscle ratios of 3.42±0.35 and 1.8±0;14 respectively (p<0.005, n = 5). Autoradiographic studies confirmed higher ^99m^Tc-B-collagelin uptake on sections of lung of mice with pulmonary fibrosis as compared to controls with heterogeneous distribution matched with that of picrosirius staining (fig 6C).

## Discussion

We have developed a specific probe for collagen which makes possible to obtain *in vivo*, specific SPECT imaging of fibrosis, using a new approach combining the advantages of the specificity of a collagen receptor, and the diffusing properties of a peptide.

Combinatorial peptide libraries are usually screened for epitope mapping such as, for example, the epitopes of antibodies directed towards vWF and to its platelet receptor GPIb [17], [18]. In the case of the anti–GPVI 9O12.2 moAb, the use of a linear phage display library [19], and of a constrained bacterial library (this study) indicated that the epitope is conformational, but cannot be used to identify a linear or discontinuous motif. Structural studies; such as X-ray analysis and/or modeling, will help to determine the epitope structure.

Another increasing application of peptide libraries is to identify peptides with specific biological activities [20], [21]. Peptides mimicking proteins of interest obtained by screening libraries *versus* blocking antibodies, designated mimotopes, are proposed as potential targets, with either therapeutic or imaging applications.

The peptide we are describing here reproduces the specificity of GPVI and thus. mimics, at least to some degree, the collagen-binding site of GPVI. It binds *in vitro* to type-I collagen with high affinity (10^−7^ M) and also to type III collagen. *Ex vivo* collagen staining on sections of rat tail tendon, which is composed of type I collagen, and of the vascular wall containing high levels of types I and III collagens, indicates that collagelin acts as a collagen probe. A GPVI-based method of isotopic imaging has been proposed [22]. I^125^/I^123^-labeled soluble recombinant GPVI-Fc was used for non-invasive imaging of vulnerable atherosclerotic plaques in ApoE−/− mice *in vivo* by SPECT, and *ex vivo* by autoradiography. However, slow blood clearance leading to high background activity and low signal-to-noise ratio was observed. GPVI-Fc, which consists of the extracellular part of GPVI fused to the IgG Fc domain [12], has a high molecular mass that prevents it from diffusing into interstitial tissues, and also has antithrombotic properties [23]. In contrast, collagelin has the advantages of being small, which facilitates its diffusion to its target, and of having no effect on platelet function.

Peptides with collagen-binding properties have previously been reported: cyclic peptides mimicking the collagen-binding site of vWF bound to rat tail collagen, and inhibited vWF binding to calfskin and human collagen, but only at high concentrations [24]; phage display also made it possible to identify the collagen-binding protein rNecH1 from *Necator americanus* [25], but no further use of these peptides or proteins has been reported. Recently, CNA35, the collagen binding part of a bacterial protein, has been successfully used as a fluorescent probe for collagen [26]. However, it cannot be used for *in vivo* imaging of organs since it does not cross the endothelial barrier [27]. Cy5.5-RGD which targets integrins on myofibroblasts and a sequence in procollagen is an indirect probe for fibrinogenesis but it does not bind to mature collagen type I or III fibers [8].

Since collagelin is a low molecular mass collagen-specific probe, we postulated that it could be used as a collagen tracer *in vivo*. The results reported here demonstrate that radiolabeled collagelin does not accumulate to any significant degree in healthy tissues or in blood, but that it clearly accumulates in post-infarct myocardial scars. This experimental model yields a clearly identifiable, dense but limited area of fibrosis, generally located at the apex of the heart. Specific accumulation of radiolabeled collagelin provided good contrast molecular imaging of the fibrotic scar, clearly distinguishable from the healthy myocardium. Images obtained by planar scintigraphy in this model, in which the scar is often thin and small in volume, indicate that collagelin represents a powerful probe for detecting fibrosis. This was confirmed by the specific accumulation of the radiotracer in lung fibrotic lesions. In this model, fibrosis that is looser than in the MI scar, was nevertheless identified by the probe.

At the time of the observation, we did not notice any accumulation of the tracer in the skin of the animals, that would have interfered with the signal of interest. We however have observed a low level of collagelin uptake in bone marrow. It thus appears there is a preferential uptake of the tracer by the fibrous collagen as compared to constitutive collagen in organs. Indeed, GPVI does not bind to monomeric collagen but interacts with a structural motif present on the cross-linked helicoidal structure of collagen [10]. The lower uptake of collagelin by the organs normally rich in collagen as compared to fibrosis, could be explained by a lower number of accessible GPVI binding sites due to fiber structure and interaction with other proteins of the matrix in this normal tissue.

Specific tracers for collagen represent a challenge and considerable interest in various imaging methods, including radionucleide imaging and MRI, is currently developing. A gadolinium-based, collagen-targeting contrast agent, EP-3533, has recently been reported to make it possible to carry out *in vivo* molecular MR imaging of fibrosis in another model of scarred infarction, in the mouse [28]. Collagelin appears to have greater affinity for collagen than EP-3533 [9]. In addition, compared to MRI, specific radionucleide imaging by SPECT or PET presents the advantages of having much higher sensitivity, and no toxicity, since a very low tracer dose (pico- or nanomolar) is sufficient to obtain an accurate target-to-background ratio. In a recent study, direct comparison of signals obtained with nanoparticles labeled with both iron oxide and Cu-64 showed that the sensitivity of PET is 50-fold higher than that of MRI [29]. This, explains why SPECT and PET imaging are undergoing considerable development worldwide, especially in malignant and cardiovascular diseases. Similar developments are also expected in other medical fields, in which the targeting of relevant processes is potentially clinically useful.

This is the first time, to our knowledge, that a mimotope of GPVI has been produced with proven *in vitro* and *in vivo* collagen-specific targeting properties. Optimizing collagelin should now be considered to improve the *in vivo* signal to noise ratio. Improved derivatives of collagelin should be developed by further increasing the affinity of the molecule for collagen, and improving the labeling procedures. Indeed, the labeled streptavidin-B-collagelin complex has the disadvantages of hepatic accumulation and a long half life. However, these difficulties could be avoided by using directly labeled collagelin, which looks very promising on the basis of our data. The circulation time of the peptide could be prolonged by a moderate increase in its size, such as, for example, by PEGylation or by constructing a dimeric peptide with increased avidity for collagen. Radiolabeling with positron emitters could also be considered for the absolute PET quantification of fibrosis *in vivo*, which would allow the non invasive therapeutic follow-up of fibrotic diseases.

Collagelin is thus a new and original collagen-specific probe with a very wide field of possible applications as a tracer of fibrotic tissues, in both non-vascular and vascular disorders.

## Material and Methods

### Screening the FliTrxTM peptide library against the anti-GPVI antibody

Anti-GPVI 9O12.2 IgGs and soluble recombinant GPVI-Fc were obtained as previously described [13]. The cyclic dodecapeptide bacterial FliTrx™ Random Peptide Display Library was from Invitrogen (San Diego, CA). Bacterial cultures and general panning methods were conducted according to the manufacturer's protocol. Briefly, the pFliTrx™ vector with the P_L_ promoter from the bacteriophage that drives expression was propagated in *E. coli* strain GI826. after induction of the expression of the thioredoxin–flagellin fusion proteins containing the peptide inserts, bacterial culture in NaCl (150 mM) containing α-methyl mannoside (10 mg.mL^−1^) and non-fat dry milk (10 mg.mL^−1^) were incubated in tissue culture plates coated with 9O12.2 IgG. After several washing steps, bound bacteria were detached and amplified before being subjected to a new round of panning. After five rounds, bacterial colonies plated on solid medium were randomly taken, amplified and induced for further identification.

### Characterization of the clones

Identification of positive clones was done by Western blotting using 9O12 IgG. Briefly, proteins of selected bacterial clones were separated by SDS-PAGE, transferred to nitrocellulose and immunoblotted with the 9O12. IgG and a phosphatase alkaline- coupled secondary antibody to mouse IgG [13]. Plasmid DNA was isolated using standard protocols, and the nucleotide sequences were determined using the FLITrx™ forward sequencing primer (5′-ATT CAC CTG ACT GAC GA-3′). The peptide sequences were deduced from DNA sequencing.

### Peptide synthesis

One peptide base on the sequence of the selected clone (underlined) [SGSGCPGRVMHGLHLGDDEGPC] was synthesized. The peptide was or was not coupled to biotin at its N-terminus *via* a short flexible spacer (SGSG), and made cyclic by disulfide bridging of the flanking Cys. The carboxyl function at the C-terminus was substituted by an amide. Peptides were prepared by NeoMPS (Strasbourg, France), and their quality controlled by HPLC and mass spectrometry.

The irrelevant, non-cyclic, biotinylated peptide P1 (SGSGVNVYAVTKENTIINPSENGD) and the cyclic peptide Pc (SGSGCGPQGARGLTGASAGGPC), biotinylated at the N–terminus (Mimotopes, Clayton Victoria, Australia) were used for control experiments.

### Binding experiments

#### Surface plasmon resonance

9O12.2 IgG was injected over biotin-peptides coupled to an SA-sensorchip in a Biacore 2000 instrument. Kinetic constants (*k*_on_, *k*_off_) were deduced at different IgG concentrations (from 1.25 to 10 µg.mL^−1^), and the dissociation constant K_D_ = *k*_off_/*k*_on_ was calculated. In some experiments, the peptide was reduced by DTT on the sensor chip before the IgG was injected. Competition experiments were performed by mixing the IgG with recombinant soluble GPVI (25 µg.mL^−1^). Collagen binding to the peptide was analyzed by injecting type-I collagen (200 µg.mL^−1^) onto the peptide-coated sensorchip. Conversely, biotin-peptides (125, 250, 500 and 1000 µg.mL^−1^) were perfused over fibrillar collagen immobilized on a CM5-sensorchip.

#### Solid phase assays

Biotin-peptides (50 µg.mL^−1^) were incubated on microtitration wells, coated with 1 µg of type-I collagen, convulxin, collagen related peptides, bovine fibrinogen, human fibronectin, rat vitronectin, murine laminin or bovine serum albumin. Bound peptides were detected using HRP-coupled extravidin and the HRP substrate O-phenyl-dianisidine.

### Histochemical analysis

Frozen sections (8 µm) of rat thoracic aorta embedded in tissue-Tek OCT were incubated with biotin-peptides (200 µg/mL) revealed using a HRP-coupled streptavidin. Sections were counter-stained with hematoxylin-eosin (Merck). Collagen was stained using picrosirius red. Sections (20 µM) of rat tail tendon were processed as above.

### *Ex vivo* and *in vivo* imaging with radiolabeled collagelin

Biotin-collagelin and biotin-control peptide, Pc, were radiolabeled as follows. Streptavidin was dissolved in 0.01% (v/v) acetic acid at a concentration of 1 mg.mL^−1^. Reactants were mixed for 1 min in the following order: first 10 µL of streptavidin followed by 4 µL of stannous pyrophosphate, 2 µL of KBH4 (10 mg.mL^−1^ in 0.1 N NaOH) and 740 MBq of Tc-99m in 50–100 µL. Thirty min later, the solution was ready for injection. Radiolabeling efficiency was measured by thin-layer chromatography, using the classical methyl-setone method High efficiency of labeling was obtained, superior to 96% at 30 min. Labeled streptavidin was mixed with B-collagelin in a molar ratio of one to four immediately before injection.

B-collagelin was directly labeled using the same procedure as above: 10 µL of peptide [2 nmol in 0.01% (v/v) acetic acid] was mixed with 740 MBq of Tc-99m for 30 min. High efficiency of labeling was obtained, superior to 96% at 30 min. In this latter case, Tc-99m was covalently complexed by disulfide bonds, ensuring the high stability of the radiolabeling

For control analyses, B-Pc was used, after radiolabeling with ^99m^Tc-streptavidin or directly by Tc-99m, using the above-described procedures.

The labeling of streptavidin and peptides was stable for at least three hours, and then decreased with 40 to 50% free technetium being detectable at 6 hours. Labeled products were injected intravenously (iv) at the dose of 70 MBq/rat.

Myocardial infarction of the left ventricle was induced in rats as follows. Male Wistar rats (CERJ, France) were housed in a controlled environment and fed with a standard rat chow. Myocardial infarction of the left ventricle was induced by ligature of the left descending coronary artery performed under general anesthesia [1 mL.kg^−1^ i.p. ketamine (Imalgene 500, Merial) and 0.5 mL.kg^−1^ i.p. xylazine (Rompun, Bayer)] and positive pressure ventilation, as described [30]. This protocol was performed under a permit from the French Veterinary Services Directorate.

Two types of control animals were used: healthy rats, and healthy rats which had undergone a simple throracotomy (sham-operated). ^99m^Tc-biotin-collagelin blood kinetics and biodistribution were investigated by counting blood samples (taken from a jugular catheter) for 6 h after i.v. injection, and organs (extracted after sacrifice) 2 h after injection in a gamma-counter, together with aliquots of the injected preparation (CobraII, Packard, Meriden, USA). *Ex vivo* myocardial labeling and isotopic imaging were performed 4 weeks after the coronary ligature (or throracotomy), when the lesions had had the time to heal.

Rat aorta and rat myocardial scar sections were incubated in the presence of ^99m^Tc-biotin-collagelin, +/−9012 moAb, or ^99m^Tc-biotin-control peptide Pc (120 MBq/ml of RPMI) for 2 h. The tissues were then rinsed 5 times for periods of 1 minute, and exposed to the gamma-camera as well as to the instant imager for quantitative autoradiography (Instant Imager, Packard) for 15 h.

For *in vivo* experiments, radiotracers (70 MBq) were administered i.v. to anesthetized animals (pentobarbital i.p. 6 mg/100 g BW, Ceva Santé Animale, France) within 2 h of radiolabeling. Scintigraphic images were obtained 0–2 h, 4 h, 6 h, 10 h and 24 h after injecting ^99m^Tc-streptavidin-biotin peptides and 0–3 h after injecting ^99m^Tc-biotinylated peptides. Planar and tomographic 1-h acquisitions were performed using a dedicated small animal γIMAGER system (Biospace Lab, Paris, France) equipped with 2 parallel low-energy high-resolution collimators (matrix 128×128, 15% energy window centered on 140 KeV). Tracer uptake in the left cardiac area was assessed visually. Two regions of interest were drawn on the scintigrams, over the heart and over the right lung. The mean activity (cpm) per pixel was determined in each region of interest. Then heart-to-lung activity ratios (HLR) were calculated on both planar and transversal tomographic images.

After sacrificing the animals, the heart was removed and frozen, then 20-micrometer thick myocardial sections were cut perpendicular to the short axis of the ventricles in a cryostat, and exposed in a radioimager for 16 h. According to calibration studies performed with activity standards of tissue-equivalent homogenates, 50 counts/mm^2^ of ^99m^ Tc-labeled tracers corresponded to ∼210 kBq/mg in autoradiography [31]. The myocardial sections used for autoradiography, and contiguous heart sections (5 µm) fixed in acetone (−20°C), were stained with hematoxylin-eosin, Masson's trichrome and picrosirius red to determine the location and extent of the fibrotic myocardial scarring.

The lung fibrosis model was realized as already described [32]. Male C57BL/6J mice, aged 6–7 weeks were kept in accordance with INSERM rules. On day 0, mice were administered 80 µg of bleomycin hydrochloride (Bleomycine Bellon, Aventis, France) intratracheally. Mortality was assessed daily over a 14 day period. Naïve mice were used as controls.

At day 14 mice received one intravenous injection of 99mTc- B-collagelin or of 99mTc- B-Pc (3 MBq). Then planar whole-body scintigraphic imaging (60 min duration) was performed 1 hour after tracer injection as above. At the end of the experiment, animals were sacrificed and lung were dissected for gamma counting, autoradiography, and histology.

### Statistical analysis

Scintigraphic and autoradiographic quantitative parameters are expressed as means±SEM. The unpaired t-test was used to compare collagelin data in 2 groups of animals (rats with myocardial scar versus sham-operated rats), or radiotracers in the rats with a myocardial scar (collagelin versus control peptide Pc). The level of significance was set at p<0.05.
